# Locum Tenencies

**Published:** 1907-06-29

**Authors:** 


### Resident Medical Officers' Department.

[Contributions to this Column are invited, and, if accepted, will be paid for.]

### LOCUM TENENCIES.

### FROM THE "LOCUM'S" POINT OF VIEW.

With the approach of the holiday season the annual summer demand is renewed for the practitioner's deputy, or "locum tenens." As a good many young medical men will probably undertake one of these posts for the first time during the next three months, we propose to offer a few remarks drawn from a varied, if not very extensive, experience. We hold very strongly that any man contemplating general practice is wise if he first accept two or three locum tenencies. Even during the very brief introductory round with his principal, and much more so when left alone with his responsibilities, he will learn a great deal that will be of value to him afterwards. All sorts of questions, hitherto undreamed of, can be studied at first hand, such as the relative advantages of horse and motor transport; of male and female dispensers; of contract appointments, club, parish, and the like.

There are broadly two classes upon which medical men depend for their deputies. One includes those who for various reasons devote their life, or at least many years of it, to this kind of work. The other is composed of young men with a little time on their hands to fill in until they can apply for some coveted vacancy in a hospital or other service, or who are taking a few months to look around them before they make up their minds to settle down. At any rate, they are casually and not regularly employed; against their inexperience of private practice, they have the advantages of keenness, energy, anxiety to learn and to please, and very often of considerable professional ability. If they have recently filled a resident appointment, they are the better equipped against those diagnostic errors into which we all stumble now and then.

Apart from the educational benefits, a locum tenency is often a most enjoyable holiday. New faces and friends, change of scene and of work are the essentials for that reinvigoration which is the test of a good holiday, and they can all be obtained by taking charge of someone's practice in an unfamiliar part of the country. The work is seldom excessive, because the practitioner chooses his slack season to leave it, and there is always a certain number of his patients who will save up their ailments for his return rather than entrust themselves to a stranger. The secret of getting through the work in time for a little relaxation afterwards lies in rigid punctuality in the morning. A locum tenens should, of course, never shirk taking pains over his duties; that is unfair to the principal: but a businesslike promptness and regularity is appreciated both by his servants and his patients, and will result in legitimate opportunities for golf, cricket, tennis, etc., in the long days of summer. It is in the months from June to September that locum work is most easily obtained and most pleasurable, and the demand then is in fact in excess of the supply; no man of good address and testimonials need lack employment at this season.

There are various ways in which the would-be locum tenens may get into communication with practitioners. Advertisements in the medical journals generally emanate from those who do locum work regularly; there are better ways for the casual class. Ex-house officers can always rely on the good offices of their visiting staff, or of the dean of the school if one is attached; former students often write to their old school for a trustworthy man, and recently qualified men who have impressed their teachers as tactful and well-mannered are fairly certain to find berths in this way. Probably the majority of men go to one or other of the medical agencies, and that also is a good plan: at this time of year a well-qualified man of unimpeachable references is welcomed by the managers, and can indeed frequently pick and choose among the applications from practitioners. There are, needless to say, all sorts and conditions of practitioner: as a rule, however, it is those of the more flourishing kind who commonly engage a locum tenens, and with whom therefore one most often comes in contact. One hears now and then of men who have had the ill-fortune to spend a month or so in some house where due allowance has not been made for the difficulties of their position; where in fact they have been treated as a sort of a servant, and even regarded as if they were likely to steal the spoons; but these cases are distinctly rare. The reception of the locum tenens will probably reflect his own behaviour; courtesy and adaptability are very soon observed and repaid by those of the household in which he is a visitor.

The relations of a locum tenens to his principal's servants are necessarily a little delicate: the latter are quick to resent any departure from their ordinary routine, and are very critical of their employer's substitute. The surest way for him to gain their respect and establish his authority is to carry out energetically the duties undertaken. They will not admit that he can be the equal of their own master, but they note keenly enough whether he is making a good attempt; the report of the coachman on the temporary doctor is soon conveyed to the coachmen, and so to the ears, of the other practitioners round about.

There are various points on which friction has been known to arise between the high contracting parties, and the arbitration of the editor of a medical paper is sometimes invoked. Thus some teetotal medical men object to providing any alcoholic beverages at meals; to these of course the deputy has no legal claim, but some practitioners go so far as even to prohibit his introducing them at his own expense. Sometimes the washing bill is the bone of contention: it is held to be the established custom in the profession that a locum should pay for his own washing, but as a matter of fact nearly all practitioners bear this expense themselves. A busy man who has just enjoyed a good holiday, and returns to find his one anxiety (that about his locum tenens) dispelled, is not apt to cavil about trifles or to treat his deputy ungenerously; still, it is well to remember that the host has a perfect right to charge washing against his locum tenens if he thinks fit. Occasionally the latter claims notification, inquest, and post-mortem fees as his own property: there is no legality about such claims; the fees are part of the earnings of the practice.

Of difficulties with cantankerous patients there are always a few. Some at every visit will inquire pointedly when "the doctor" returns; others demand to know whether one is legally qualified. Most will criticise severely any proceeding, suggestion, method of examination, etc., which is not familiar to them under the regular *régime*. But, taking them all round, there is much less trouble than one would naturally expect; so considerate are some patients for their new medical attendant, that they give the impression of rather enjoying a holiday from the old one. This is especially the case with housefast sufferers from chronic valvular disease and similar maladies, to whom a new face is somewhat of an event, and a new doctor of more benefit than much physic. Many of these people do actually improve in the most wonderful way during their doctor's absence, especially if his substitute is cheery and optimistic: the first time he notices this, the locum tenens is apt to plume himself on his superior treatment, but probably the other reason we have given is usually the truer one. There are, however, occasions when a locum tenens who brings a fresh and unbiassed mind to bear on a case may detect that which has escaped the notice of the practitioner. In such an event the former need not hesitate to alter the line of treatment if necessary, provided he is sure of his ground; his duty to his employer includes that of the latter to his patients. But he is well advised to abstain from radical changes except on clear evidence of their necessity, and to be very tactful in his dealings with such cases.

---

[The Editor will be glad to consider contributions from the opposite point of view, that of the practitioner on the subject of his locum tenens; one (or more) of these will be printed in the practitioner's page later on in the holiday season.]

